# Immunoinformatics-guided design of a multiepitope peptide vaccine targeting the receptor-binding domain of SARS-CoV-2 spike glycoprotein: insights from Indonesian samples

**DOI:** 10.1515/jib-2024-0025

**Published:** 2025-01-14

**Authors:** Irvan Faizal, Darrian Chandra, Tarwadi, Sabar Pambudi, Astutiati Nurhasanah, Rizky Priambodo, Muhammad Yusuf

**Affiliations:** Research Centre for Vaccine and Drugs, National Research and Innovation Agency-Republic of Indonesia, Kawasan Puspiptek Serpong, Tangerang Selatan 15314, Indonesia; Department of Biotechnology, Faculty of Biotechnology, Atma Jaya Catholic University of Indonesia, BSD Campus, Tangerang 15345, Indonesia; Department of Biology, Universitas Negeri Jakarta, Jl. Rawamangun Muka, Rawamangun Jakarta Timur 13220, Indonesia; Research Center for Molecular Biotechnology and Bioinformatics, Universitas Padjadjaran, Bandung 40133, Indonesia; Department of Chemistry, Faculty of Mathematics and Natural Sciences, Universitas Padjadjaran, Jatinangor 45363, Indonesia

**Keywords:** SARS-CoV-2, variants, spike protein, receptor binding domain, immunoinformatic, epitope vaccine candidate

## Abstract

The emergence of new variants of SARS-CoV-2, including Alpha, Beta, Gamma, Delta, Omicron variants, and XBB sub-variants, contributes to the number of coronavirus cases worldwide. SARS-CoV-2 is a positive RNA virus with a genome of 29.9 kb that encodes four structural proteins: spike glycoprotein (S), envelope glycoprotein (E), membrane glycoprotein (M), and nucleocapsid glycoprotein (N). These proteins are vital for viral activity, with the S protein facilitating attachment and membrane fusion through the receptor-binding domain (RBD) located in the S1 subunit. The RBD recognizes and binds to the human angiotensin-converting enzyme 2 (ACE-2) protein. An immunoinformatic-aided design of a peptide-based multiepitope vaccine candidate targeting the RBD glycoprotein is constructed from the SARS-CoV-2 sequence data base from various regions of Indonesia (Jakarta, West Java, and Bali). The results show that the RBD region of with accession ID EPI_ISL_15982641 from West Java had the highest antigenicity of 0.5904. This isolate is non-toxic and non-allergenic and shows a total of 18 LBL epitopes, 72 CTL epitopes, and 98 HTL epitopes. The epitope that has the best overall binding affinity was GCHNKCAY for MHC-I and GGCVFSYVGCHNKCAYWV for MHC-II which show a binding affinity of −13.6 and −15.5 (kcal/mol), respectively. Therefore, this study aims to design an epitope vaccine candidate based on samples from Indonesia that has good characteristics and may have the potential to stimulate an immune response against SARS-CoV-2.

## 1 Introduction

The global impact of COVID-19 has led to a significant increase in research and development efforts to combat the virus. With the emergence of new variants, it has become even more crucial to develop effective vaccines and therapies to control the spread of the disease. Several vaccines have been developed and are being used worldwide, but the virus is still spreading in many regions, and new variants are emerging, highlighting the need for continued research and development. The immunoinformatic approach for designing epitope-based vaccines has become an increasingly popular strategy in recent years. This approach involves using computational tools to predict potential T cell epitopes that can stimulate an immune response against the virus. In addition to being an efficient and cost-effective method, it also allows for a more targeted and personalized approach to vaccine development. Furthermore, the epitope-based vaccine approach has several advantages over conventional vaccine strategies. It enables the design of highly specific and selective vaccines that are less likely to trigger unwanted immune reactions or side effects. It also allows for the rapid development of vaccines against new and emerging virus strains.

The emergence of new variants of SARS-CoV-2, including the Alpha, Beta, Gamma, Delta, Omicron variants and XBB sub-variants, has contributed to the rising number of coronavirus cases worldwide SARS-CoV-2 has a positive viral RNA genome that expresses open reading frames coding for structural and non-structural proteins, such as the Spike protein, Ribosome Binding Site, Nucleocapsid proteins, Envelope proteins, membrane proteins, and ORF1a proteins. The Spike (S) protein is responsible for CoV entering the host cells and binding to its receptor, angiotensin-converting enzyme 2 (ACE2) [1]. The S protein is the main immunogenic component of the virus and is often used in vaccine development [2]. A Receptor Binding Domain (RBD) is present in the S1 subunit of the S protein, which recognizes and binds to the human ACE2 protein [3]. The spike protein is a large, trimeric glycoprotein that protrudes from the virus’s surface. It plays a crucial role in the virus’s ability to enter host cells by recognizing and binding to the host cell receptor through its receptor-binding domain (RBD). Specifically, the RBD of the spike protein binds to the ACE2 receptor on the host cell’s surface and undergoes conformational changes to expose itself for binding. This highly specific interaction, involving multiple contact points between the RBD and ACE2, facilitates strong binding. This binding then triggers structural changes in the spike protein, allowing the S2 subunit to mediate the fusion of the viral envelope with the host cell membrane.

There are numerous approaches to designing vaccine candidates against SARS-CoV-2, one of which is the immunoinformatic approach of designing epitope or multiple-epitope peptide-based vaccine candidates [4]. Antigenic peptides for T cell activation are generally administered via major histocompatibility complex (MHC-I) and MHC-II [4], 5], so prediction of T cell epitopes is typically conducted by predicting MHC epitopes. The development of COVID-19 vaccines utilized several innovative approaches, including mRNA vaccines that employ synthetic messenger RNA to produce the viral spike protein. The mRNA technology enabled rapid design and production, reducing development timelines and allowing quick adaptation for large-scale production [6]. This method avoids viral vectors, reducing pre-existing immunity issues, differs from protein subunit vaccines by producing antigens *in vivo*, and enhances safety and simplicity by not using live or inactivated viruses. The mRNA vaccines were swiftly authorized, crucial for widespread immunization and reducing COVID-19’s impact, and allow for rapid updates to address emerging variants [7].

In this study, we employed an immunoinformatic approach, wherein we collected SARS-CoV-2 sequence data from diverse regions of Indonesia, including Jakarta, West Java, and Bali. Our primary objective was to formulate a potential vaccine candidate based on multiple epitopes derived from the whole genome sequences of the Indonesian virus. We utilized various computational tools and databases to predict potential T cell epitopes and assess their antigenicity, toxicity, and allergenicity. The identified epitopes were then utilized to construct a vaccine candidate, which underwent further evaluation to determine its immunogenicity and efficacy in eliciting an immune response against SARS-CoV-2. The overarching goal of this research is to make a significant contribution to the development of a safe and effective vaccine against COVID-19, with a particular focus on Indonesia.

## 2 Materials and methods

This comprehensive study was conducted over six months, from July 2022 to January 2023, and comprised six distinct stages. The initial stage involved gathering the SARS-CoV-2 sequence data base from various regions of Indonesia (Jakarta, West Java, and Bali) using GISAID’s EpiCov database (https://doi.org/10.55876/gis8.240513uc). Subsequently, the sequences were aligned, and a detailed phylogenetic analysis was carried out to determine the genetic relationship between the different viral strains. Antigenicity, allergenicity, and toxicity analysis were then performed on the aligned sequences to evaluate their potential for use in vaccine development. The next phase of the study involved predicting the T and B cell epitopes through the utilization of advanced tools and databases. Finally, the epitopes were analyzed for protein interaction and visualized using GalaxyPepDock software to gain a deeper understanding of the protein structures.

### 2.1 Software

The following software applications were utilized throughout the study: MEGA X, available at https://www.megasoftware.net, Allertop, available at https://www.ddg-pharmfac.net/AllerTOP/method.html, ToxiBTL, available at https://server.wei-group.net/ToxIBTL/home.html, BepiPred, available at https://services.healthtech.dtu.dk/service.php?BepiPred-3.0, Immune Epitopes Database (IEDB), which was accessed at http://tools.iedb.org/processing/ for MHC-I and http://tools.iedb.org/mhcii/ for MHC-II, GalaxyPepDock, available at https://galaxy.seoklab.org/, and PRODIGY, accessible at https://wenmr.science.uu.nl/prodigy/.

### 2.2 Methods

#### 2.2.1 Collection and selection of SARS-CoV-2 sequences database in Indonesia

To create a thorough database of SARS-CoV-2 sequences in Indonesia, we obtained whole genome sequences from GISAID’s EpiCov database (https://doi.org/10.55876/gis8.240513uc). The parameter for selecting the sequence data used were: Complete genome, High coverage, and Location in Indonesia. These parameters were used to obtain the most complete sequence from Indonesia that can be used for further processing. We collected a total of 15 samples from each of the regions of Jakarta, West Java, and Bali, consisting of 5 Delta, 5 Omicron, and 5 XBB variants. In our subsequent analyses, we utilized the reference genome of SARS-CoV-2 Wuhan (WIV04, https://gisaid.org/wiv04/) to maintain consistency and comparability.

#### 2.2.2 Alignment of receptor-binding domain (RBD) sequence of SARS-CoV-2

The collected sequences were aligned using the ClustalW method in MEGA X. The region of interest was the RBD from positions 319 to 591, which was then translated into its corresponding protein sequence.

#### 2.2.3 Constructing a phylogenetic tree and determining genetic relationships

The aligned sequence data obtained from MEGA X was utilized to construct a phylogenetic tree, employing the Neighbour-joining method. The collected data was then examined to determine the genetic relationships between the samples.

#### 2.2.4 Analysis of antigenicity, allergenicity, and toxicity of SARS-CoV-2 protein sequences

The antigen results that have been obtained are then validated to make sure again that the antigen is able to cause an immune response. This is done by predicting the epitope sequence that will be generated from the antigen candidate. The protein sequences obtained from the sequence alignments were analyzed for their antigenicity, allergenicity, and toxicity using specialized tools. The antigenicity of the proteins was calculated using VaxiJen, while AllerTop was used to determine the allergenic potential of the proteins. Additionally, the toxicity of the proteins was assessed using ToxIBTL.

#### 2.2.5 Predicting epitopes of T and B cells in SARS-CoV-2

The epitope is expected to be recognized by T cells (Cytotoxic T Lymphocyte or CTL and Helper T Lymphocyte or HTL) and B cells (Linear B Lymphocyte or LBL). CTL epitopes were predicted using IEDB MHC I (http://tools.iedb.org/mhci/), with prediction method parameter settings: ANN 4.0, allele: select HLA allele reference set, length of epitope: 9 mer. IC 50 was used as a screening to determine the strength of the resulting epitope. HTL epitopes were predicted using Immune Epitope Database (IEDB) MHC II (http://tools.iedb.org/mhcii/), with prediction method parameter settings: NN-align 2.3, allele: select full HLA allele reference set, length of epitope: 15 mer. LBL epitopes were predicted using BepiPred 2.0 (https://services.healthtech.dtu.dk/service.php?BepiPred-2.0) with threshold: 0.5, a total of 18 LBL epitopes were successfully predicted from the RBD sequences of vaccine candidates [8].

#### 2.2.6 3D visualization and binding affinity calculation of epitopes

The selected epitope is then subjected to protein – receptor docking to ensure that the epitope resulting from antigen cutting will be able to bind to the MHC receptor. Since CTL epitope will be presented by MHC I receptor while HTL epitope will be presented by MHC II receptor, CTL epitope will be docked with MHC I (Protein Data Bank, PDB ID: 1XR8) and HTL epitope with MHC II (PDB ID: 6BIY). Docking is done using GalaxyPepDock webserver (https://galaxy.seoklab.org/cgi-bin/submit.cgi?type=PEPDOCK) [9]. The docking result (.PDB) file was then analyzed for binding affinity or Gibbs free energy Δ*G* (kcal/mol) using PRODIGY webserver (https://wenmr.science.uu.nl/prodigy/), and then compared with the binding affinity value of the epitope versus the binding affinity of the known control protein. This is an important thermodynamic parameter to assess whether the binding affinity in a biological complex is energetically feasible or not. Usually, negative values indicate the greater likelihood of biomolecules to form a biologically plausible complex.

## 3 Result and discussion

### 3.1 Phylogenetic analysis

**Figure 1:**
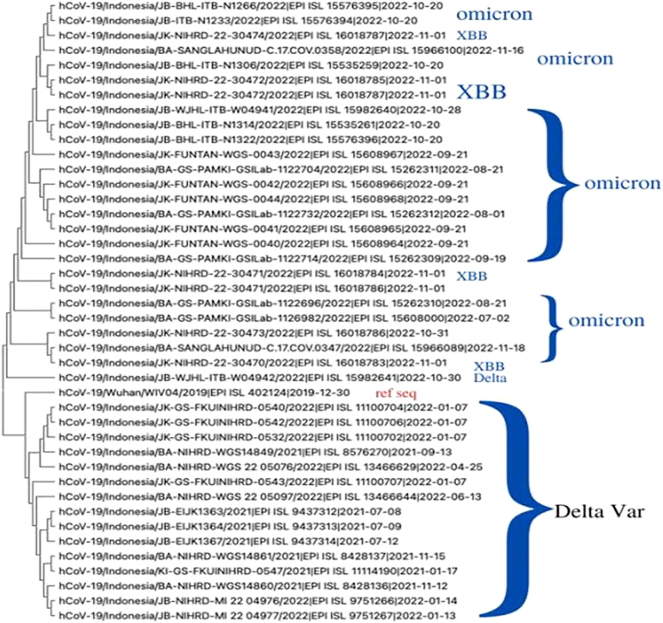
Phylogenetic analysis of whole genome sequencing of Delta, Omicron, and XBB variants from Jakarta (virus name coded by JK), West Java (JB), and Bali (BA).

In this study, samples of positive cases of SARS-CoV-2 were collected from various regions in Indonesia, including Jakarta (coded as JK), West Java (JB), and Bali (BA), spanning the period from January 2021 to January 2023. This extensive timeframe offered a comprehensive overview of the virus’s spread over nearly two years. Genomic data analysis involved conducting a phylogenetic study based on whole genome sequencing. The results of this analysis revealed that the reference gene (WIV04) closely clustered with a clade of Delta variants. Figure 1 illustrates the phylogenetic tree of Delta, Omicron, and XBB variants in Indonesia, showing that WIV04 shares a common lineage with the Delta variants situated above it. The shorter branch length between WIV04 and the Delta variants implies a close genetic relationship. Conversely, the Omicron variants formed a distinct clade beneath the Delta variants. Within the Omicron variants, one sample (accession ID EPI_ISL_15982641) formed a monophyletic group with WIV04, displaying a shorter branch length compared to other samples, indicating a close genetic affinity to WIV04. These findings offer valuable insights into the evolutionary dynamics and dissemination of SARS-CoV-2 in Indonesia, providing essential information for public health strategies aimed at pandemic control.

The phylogenetic tree depicting Delta, Omicron, and XBB variants in Indonesia highlights the prevalence of the Delta variant in the country, evidenced by the largest number of samples within the Delta clade. In contrast, the Omicron variant forms a separate clade with fewer samples, while the XBB variant constitutes a distinct outgroup. The extended branch length of the XBB variant suggests a more distant relationship with both Delta and Omicron variants. The geographical distribution of these samples is mirrored in the phylogenetic tree, with Bali and Jakarta samples clustering together in specific clades. In contrast, samples from West Java are dispersed across the tree, indicating potential regional disparities in the prevalence and distribution of different SARS-CoV-2 variants in Indonesia, as suggested by [10]. Overall, this phylogenetic analysis offers crucial insights into the genetic relationships among various SARS-CoV-2 variants in Indonesia, emphasizing the ongoing need for vigilant surveillance and monitoring of viral evolution and transmission, as emphasized by [11].

### 3.2 Evaluation of antigenicity, allergenicity, and toxicity of Omicron and Delta variant from Jakarta, West Java, and Bali

Antigenicity score is a measure of how well antibodies produced by the human immune system can recognize and neutralize viruses. The higher the antigenicity score, the more effective antibodies are against viruses. The SARS-CoV-2 virus has a spike protein that acts as a key to enter human cells by binding to the ACE2 receptor on the cell surface. This spike protein is also the main target of antibodies produced by vaccines or previous infections. The omicron and delta variants have some mutations on the spike protein, especially on the part called the receptor binding domain (RBD), which makes it easier to bind to the ACE2 receptor and harder to be recognized by antibodies. These mutations change the shape and properties of the spike protein, making antibodies that are effective against the wildtype virus less effective against the new variants. In this study, the delta variant had a lower antigenicity score than the wildtype virus. Therefore, the SARS-CoV-2 wildtype virus (EPI_ISL_402124) has a higher antigenicity score than the omicron (EPI_ISL_15982640) and delta variants (EPI_ISL_8576270 and EPI_ISL_11114190) because its spike protein is more easily recognized and neutralized by antibodies produced by the human immune system.

Analysis results, presented in Table 1 via VaxiJen v2.0, unveil the antigenic potential of diverse viral isolates, all surpassing the threshold (>0.40). Particularly noteworthy, accession ID EPI_ISL_15982640 displayed the highest antigenicity score at 0.5094, indicating its robust antigenic nature and promise as a viable vaccine candidate. A striking revelation in this study is the higher antigenicity scores of Omicron variants compared to Delta variants. Recent research attributes this difference to an increase in nonself short constituent sequences (SCSs) in the RBD. This change results in reduced virulence and a heightened affinity to ACE2, explaining Omicron’s elevated antigenicity and decreased virulence [12]. Omicron’s enhanced binding affinity for ACE2, due to numerous nonself mutations in the RBD, underscores its potential for increased transmission. However, epitope allergenicity presents a significant challenge in vaccine development. Rigorous verification procedures were employed to confirm the non-allergenic nature of identified epitopes. Utilizing AllerTop and ToxIBTL tools, protein sequences of the vaccine were analyzed to assess the likelihood of eliciting allergic reactions or toxicity. Encouragingly, all viral isolates analyzed in this study showed no potential for causing allergic reactions or toxicity, a crucial factor in vaccine development [13]. The outcomes of these analyses provide invaluable insights into vaccine candidates’ antigenic potential, allergenicity, and toxicity, fundamental considerations in the meticulous process of developing vaccines for infectious diseases.

**Table 1:** Antigenicity score, allergenicity, and toxicity of Omicron and Delta variant from Jakarta, West Java, and Bali.

Accession ID	Region	Variant	Antigenicity score	Antigenicity	Allergenicity	Toxicity	
EPI_ISL_15982640	West Java	Omicron	0.5094	Antigenic	Nonallergen	Nontoxic	This study
EPI_ISL_15262309	Bali	Omicron	0.4837	Antigenic	Nonallergen	Nontoxic	
EPI_ISL_8576270	Bali	Delta	0.4858	Antigenic	Nonallergen	Nontoxic	
EPI_ISL_15608966	Jakarta	Omicron	0.4996	Antigenic	Nonallergen	Nontoxic	
EPI_ISL_11114190	Jakarta	Delta	0.4934	Antigenic	Nonallergen	Nontoxic	
EPI_ISL_402124 (ref gene)	Wuhan	Wildtype	0.662	Antigenic	Nonallergen	Nontoxic	[14]

### 3.3 T and B cell prediction

The importance of the antigenicity and T- and B-cell epitope count characteristics of selected RBD candidates cannot be overstated in the quest for designing effective vaccines against SARS-CoV-2. The receptor-binding domain (RBD), a component of the spike protein, plays a pivotal role as it binds to the human ACE2 receptor, facilitating viral entry into host cells. Additionally, the RBD serves as a prime target for neutralizing antibodies and T-cell responses, making it a key focus in vaccine development. The identification and prediction of T and B cell epitopes, which vary in length from 7 to 20 residues, are critical steps in this process [15]. Regarding the RBD serving as a prime target for neutralizing antibodies, in the initial strains of SARS-CoV-2, the RBD was relatively conserved, making it effective for targeting by antibodies. Early vaccines and treatments were based on this stable RBD structure. However, as the virus evolved, variants of concern (VOCs) with mutations in the RBD emerged. These mutations can change the RBD structure and affect its binding to the ACE2 receptor. Variants like Delta [16] and Omicron [17] have shown RBD changes that impact how antibodies from earlier strains recognize and neutralize these new variants. Despite these mutations, the RBD remains a key target for neutralizing antibodies. The effectiveness of antibodies against variants can vary, and some mutations may reduce antibody binding efficacy. This makes continuous monitoring and adaptation of vaccine formulations necessary.

Predicting T-cell epitopes holds particular significance as it helps identify the smallest peptide within an antigen capable of stimulating CD4 or CD8 T-cells, thereby generating immunogenicity. Central to this prediction is the binding of peptides to the major histocompatibility complex (MHC), making these binders essential for T-cell activation. Accurate prediction of MHC binders is pivotal for efficient vaccine design because of their importance in activating the immune system’s T-cells. The experimentally determined B- and T-cell epitopes of SARS-CoV-2, crucial data in this context, were sourced from the publicly available IEDB. These epitopes were meticulously chosen for further analysis, ensuring they met specific criteria, including positive B-cell assays, positive T-cell assays, and positive MHC binding assays.

In this study, a comprehensive combinatorial screening approach was employed, encompassing the entire range of predicted B-cell and T-cell epitopes (MHC-I and MHC-II) across diverse lengths and protein sequences. The primary objective was to identify immunogenic segments that could function as dual-purpose B-cell and T-cell epitopes. Through meticulous comparative evaluation, epitopes demonstrating 100 % sequence coverage were precisely pinpointed, considering the selection of immunogenic region lengths aimed at achieving maximum coverage of either B-cell or T-cell epitopes within the mapped regions. This rigorous analysis and selection process served as the cornerstone for the strategic design of vaccines against SARS-CoV-2, utilizing the insights derived from these immunogenic segments to enhance the efficacy of future vaccine candidates.

In the course of this research, a specific viral strain with accession ID EPI_ISL_15982640, exhibiting the highest antigenicity score, was scrutinized. Analysis from the IEDB revealed 18 LBL epitopes with potential indications for inducing B cells. Conversely, T-cell epitope prediction was conducted to assess the spike protein’s ability to stimulate CD8+ CTL and HTL. CTL epitopes were forecasted through screening for epitopes capable of binding to MHC class I, a macromolecule facilitating the presentation of foreign antigens to CTL. This study identified 72 CTL epitopes, crucial in vaccine development as they prompt the production of CD8+ T cells, which can identify and eradicate infected cells. Additionally, 98 HTL epitopes were discovered in this study, predicted based on the foreign antigens’ ability to bind with MHC-II (Table 2). These findings underscore the chosen candidate antigen’s capability to stimulate both B cells and T cells, thereby initiating the body’s immune system activation.

**Table 2:** Antigenicity and T- and B-cell epitope count characteristics of selected RBD candidate.

Antigen	Antigenicity	Number of epitope			
		LBL	CTL	HTL	
EPI_ISL_15982640 (candidate)	0.5094	18	72	98	This study
EPI_ISL_402124 (ref gene)	0.4672	17	2	10	[18]

Subsequently, each of these epitopes underwent meticulous assessments for antigenicity, allergenicity, toxicity, and comparison with human amino acid sequences. Antigenicity prediction was revisited, utilizing a threshold of 0.4, and allergenicity was verified to ensure that the epitope would not induce allergic reactions, generating cytokines for allergies instead of those for immune responses. The prediction of HTL epitopes holds paramount importance in vaccine development, as these epitopes are responsible for triggering the production of CD4+ T cells, activating both B cells and CD8+ T cells [19]. Together, these cells collaborate to eradicate the pathogen and provide enduring protection against future infections.

The binding affinity between a virus and its host receptor is a critical factor that determines the ability of the virus to infect host cells. In the case of SARS-CoV-2, the spike protein on the surface of the virus binds to the human angiotensin-converting enzyme 2 (hACE2) receptor on the surface of host cells to initiate infection. The binding affinity of the spike protein to the hACE2 receptor can be measured in terms of the free energy released upon binding [20], which is expressed in units of kcal/mol. A lower free energy value (more negative) indicates a stronger binding affinity between the two molecules. The binding affinity value of MHC-I and II epitope of Omicron variant is a measure of how well the peptide fragments derived from the Omicron variant of SARS-CoV-2 can bind to the MHC-I and II molecules and stimulate an immune response. The higher the binding affinity, the more likely the peptide is to be recognized by T cells and trigger an immune response. In the case of the Omicron variant, it has been reported that the binding affinity of the spike protein to hACE2 is −11.8 kcal/mol [12].

In this study, the epitope that has the best overall binding affinity was GCHNKCAY for MHC-I and GGCVFSYVGCHNKCAYWV for MHC-II which show a binding affinity of −13.6 and −15.5 kcal/mol, respectively. The average binding affinity for CTL epitopes and HTL epitopes in Table 3, has binding affinity of −13.88 and −12.48 kcal/mol, respectively. The table below clearly illustrates that certain epitopes produced exhibit more negative Δ*G* values, signifying their superior binding capability compared to the control. In simpler terms, this suggests that the candidate antigen has the potential to generate epitopes with strong binding affinity for both MHC class I and II.

**Table 3:** Binding affinity value of MHC-I and II epitope of Omicron variant from West Java.

MHC-I epitope	Binding affinity (kcal/mol)	MHC-II epitope	Binding affinity (kcal/mol)	Reference
DNLLEILQKEKVNIN	−12.3	SANIGCNH	−10.3	This study
AYWVPRASANIGCN	−14.9	GCHNKCAY	−13.6	
GCVFSYVGCHNKCAY	−14.4	TGVVGEGS	−13.1	
GCVFSYVGCHNKCAYWV	−12.1	GSEGLNDN	−12.8	
GGCVFSYVGCHNKCAYWV	−15.5	EILQKEKV	−12.2	
CVFSYVGCHNKCAYWVPR	−14.1	PRASANIGC	−12.9	
**Average**	−13.88	Average	−12.48	
GCHNKCAY	−13.6	GGCVFSYVGCHNKCAYWV	−15.5	[12]

The binding affinity value of MHC-I and II epitope of Omicron variant is not a fixed number, but rather a range or a distribution that depends on various factors, such as the peptide length, the MHC allele, the prediction method, and the experimental validation. The cut-offs for MHC class I and II binding predictions are provided by IEDB. For MHC class I predictions, a percentile rank of ≤1 % or a binding affinity (IC50) threshold of 500 nM are recommended to identify peptide binders recognized by T cells. For MHC class II predictions, a consensus percentile rank of the top 10 % or a binding affinity (IC50) threshold of 1000 nM are recommended to select predicted binders. These thresholds may vary depending on the specific application and the number of peptides needed for further analysis.

This Δ*G* value in Table 3 is a relatively strong binding affinity, indicating that the Omicron variant in this study is highly infectious. CTL epitopes are the epitopes that are presented on the surface of infected cells and recognized by CD8+ T cells, while HTL epitopes are recognized by CD4+ T cells. The average binding affinity for both CTL and HTL epitopes was found to be lower than the binding affinity of the spike protein to hACE2, suggesting that the spike protein has a stronger binding affinity for hACE2 than for T cell epitopes. However, the Omicron variant was found to have higher binding affinity for both CTL and HTL epitopes than other variants. This is likely due to the mutations in the receptor-binding domain of the spike protein, which have been shown to enhance the binding affinity between the spike protein and hACE2 [21]. These mutations may also affect the binding affinity of the spike protein for T cell epitopes, which could have implications for vaccine development and immune recognition of the virus.

### 3.4 3D structure and docking analysis of potential MHC-I and MHC-II epitopes

**Figure 2:**
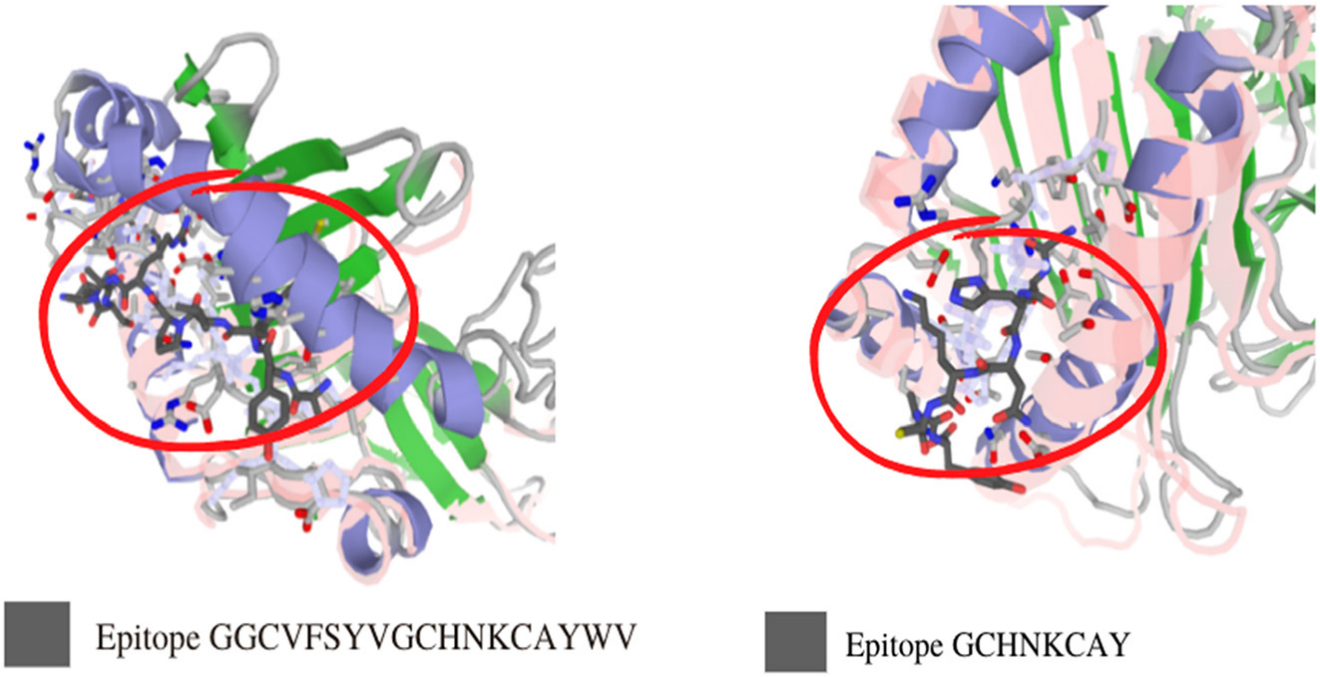
3D structures of MHC-I and MHC-II epitope of Omicron variant from West Java.

The correlation among the protein structure antigenicity, epitope prediction, accessibility, and flexibility within 3D structure was determined through GalaxyPepDock. Once potential epitopes were identified, we further analyzed them by developing 3D structures and performing docking analysis with their respective MHC alleles for GGCVFSYVGCHNKCAYWV (MHC-I epitope) and GCHNKCAY epitope (MHC-II epitope), respectively. By displaying the 3D structure and tertiary structures of selected MHC-1 and MHC-II epitopes, we were gain insights into the structural features of these peptides and how they interact with their respective MHC molecules (Figure 2). Docking analysis allows us to predict the binding affinity and orientation of the epitope within the MHC binding groove, which can provide valuable information about the nature of the interaction between the epitope and the MHC molecule [22]. Overall, by developing 3D structures and performing docking analysis with MHC alleles, we gain important insights into the structural features and binding interactions of potential epitopes, which can help to guide the development of effective vaccines.

## 4 Conclusions

In conclusion, our comprehensive analysis of SARS-CoV-2 genomic data across diverse regions of Indonesia, spanning from January 2021 to January 2023, has unveiled a highly promising vaccine candidate within the RBD region of West Java. This region not only boasts the highest antigenicity but also exhibits non-toxic and non-allergenic properties, accompanied by an extensive repertoire of 18 LBL epitopes, 72 CTL epitopes, and 98 HTL epitopes. Notably, the top-binding epitopes, GCHNKCAY for MHC-I and GGCVFSYVGCHNKCAYWV for MHC-II, demonstrated remarkable binding affinities. These epitopes serve as the bedrock for an encouraging vaccine candidate, rigorously evaluated for its immunogenicity and effectiveness against SARS-CoV-2. This research represents a critical stride toward the development of a safe and efficacious COVID-19 vaccine, with a special emphasis on addressing the unique challenges faced in Indonesia.

## List of abbreviations

ACE-2: angiotensin-converting enzyme 2
CTL: cytotoxic T lymphocytes
E: envelope glycoprotein
HTL: helper T lymphocytes
M: membrane glycoprotein
MHC-I: major histocompatibility complex class I
MHC-II: major histocompatibility complex class II
N: nucleocapsid glycoprotein
RBD: receptor-binding domain
S: spike glycoprotein
SARS-CoV-2: severe acute respiratory syndrome coronavirus 2
